# Directly Measured vs. Calculated Low-Density Lipoprotein Cholesterol Does Not Identify Additional Individuals With Coronary Artery Disease and Diabetes at Higher Risk of Adverse Events: Insight From a Large Percutaneous Coronary Intervention Cohort in Asia

**DOI:** 10.3389/fcvm.2022.932878

**Published:** 2022-07-07

**Authors:** Boqun Shi, Hao-Yu Wang, Jinpeng Liu, Zhongxing Cai, Chenxi Song, Lei Jia, Dong Yin, Hongjian Wang, Ke-Fei Dou, Weihua Song

**Affiliations:** ^1^Cardiometabolic Medicine Center, Department of Cardiology, Fuwai Hospital, National Center for Cardiovascular Diseases, Chinese Academy of Medical Sciences and Peking Union Medical College, Beijing, China; ^2^Coronary Heart Disease Center, Department of Cardiology, Fuwai Hospital, National Center for Cardiovascular Diseases, Chinese Academy of Medical Sciences and Peking Union Medical College, Beijing, China; ^3^State Key Laboratory of Cardiovascular Disease, Beijing, China

**Keywords:** lipids, lipoproteins, cardiac disease, LDL-cholesterol, percutaneous coronary intervention

## Abstract

**Background:**

The objective of our study was to assess whether calculated low-density lipoprotein cholesterol (LDL-C) is inferior to direct LDL-C (dLDL-C) in identifying patients at higher risk of all-cause mortality, recurrent acute myocardial infarction (AMI), and major adverse cardiovascular event (MACE).

**Methods:**

A total of 9,751 patients with coronary artery disease (CAD) undergoing percutaneous coronary intervention (PCI) in the Fuwai PCI registry were included. DLDL-C was measured by the selective solubilization method (Kyowa Medex, Tokyo, Japan). Correct classification was defined as the proportion of estimated LDL-C in the same category as dLDL-C based on dLDL-C levels: less than 1.4, 1.4–1.8, 1.8–2.6, 2.6–3.0, and 3.0 mmol/L or greater.

**Results:**

Underestimation of LDL-C was found in 9.7% of patients using the Martin/Hopkins equation, compared with 13.9% using the Sampson equation and 24.6% with the Friedewald equation. Cox regression analysis showed compared the correct estimation group, underestimation of LDL-C by the Martin/Hopkins equation did not reduce all-cause mortality (HR 1.26, 95% CI: 0.72–2.20, *P* = 0.4), recurrent AMI (HR 1.24, 95% CI: 0.69–2.21, *P* = 0.5), and MACE (HR 1.02, 95% CI: 0.83–1.26, *P* = 0.9). Similarly, the overestimated group did not exacerbate all-cause mortality (HR 0.9, 95% CI: 0.45–1.77, *P* = 0.8), recurrent AMI (HR 0.63, 95% CI: 0.28–1.44, *P* = 0.3), and MACE (HR 1.07, 95% CI: 0.86–1.32, *P* = 0.6). The results of the diabetes subgroup analysis were similar to those of the whole population.

**Conclusion:**

Compared with dLDL-C measurement, misclassification by the Martin/Hopkins and Sampson equations was present in approximately 20% of patients. However, directly measured vs. calculated LDL-C did not identify any more individuals in the PCI population with increased risk of all-cause mortality, recurrent AMI, and MACE, even in high-risk patients such as those with diabetes.

## Introduction

Total cholesterol (TC), low-density lipoprotein cholesterol concentration (LDL-C), hypertension, diabetes mellitus, and current smoking are all major risk factors for coronary artery disease (CAD) (1, 2). LDL-C lowering with lifestyle modification, statins, and other treatments, including ezetimibe or PCSK9 inhibitors, has resulted in significant adverse outcomes reductions (3–5). Optional LDL-C targets are <1.8 mmol/L(2) or 1.4 mmol/L(1) in CAD participants. As a result, as previously stated, having an accurate direct LDL-C (dLDL-C) essay is critical (6–8).

To obtain LDL-C concentration, ultracentrifugation of plasma at its density for 18 h is required. This procedure is time-consuming and labor-intensive (9, 10). Subsequently, the Friedewald formula, which assumes a fixed factor for the ratio of triglycerides (TG) to very-low-density lipoprotein cholesterol (VLDL-C), is generally used to estimate LDL-C (11). However, the Friedewald equation is inaccurate if the patient has hyperglyceridemia (TG ≥4.5 mmol/L) (12). The Martin/Hopkins formula estimates LDL-C using a modifiable factor for the TG/VLDL-C ratio and is expected to improve Friedewald when predicting measured LDL-C (13). Sampson formula for calculating LDL-C concentration has also been developed (14). Direct homogeneous LDL-C assays for quickly testing a large number of samples without pretreatment were developed, with results designed to be nearly identical to LDL-C values obtained following ultracentrifugation. The accuracy of these dLDL-C methods, especially when employed in individuals with severe dyslipidemia, remains controversial (15, 16). The Chinese guideline for managing dyslipidemia in adults recommends routine use of the direct method for measuring LDL-C (17), while the National Lipid Association recommends the Martin/Hopkins equation (18). The 2017 American College of Endocrinology guidelines for the management of dyslipidemia suggests that LDL-C can be estimated by the Friedewald equation but should be directly measured in patients with TG ≥2.8 mmol/L, diabetes or known vascular disease (Grade C; BEL 3) (19).

In this all-comer real-world cohort of 9,751 participants from the Fuwai PCI registry, we assessed whether calculated LDL-C using the Martin/Hopkins, Sampson, and Friedewald equations is inferior to directly measured LDL-C in identifying patients at higher risk of all-cause mortality, recurrent acute myocardial infarction (AMI), and major adverse cardiovascular event (MACE). We also assessed whether LDL-C should be directly measured in specific high-risk individuals such as those with diabetes.

## Materials and Methods

### Patient Population

A total of 10,724 consecutive patients who underwent percutaneous coronary intervention (PCI) for CAD were prospectively included from Fuwai Hospital, National Center for Cardiovascular Diseases, Beijing, China, between January 2013 and December 2013. Exclusion criteria were no direct measurement of LDL-C in the present study. Finally, there were no missing dLDL-C values in 9,751 patients. Our dedicated PCI registry by independent research personnel systematically and prospectively collected demographic and clinical characteristics, angiographic and procedural information, and follow-up data. The study was approved by the Fuwai Hospital Ethics Committees and was conducted according to the Declaration of Helsinki. Written informed consent was obtained from all participants.

### Patient Follow-Up

After index PCI, patients were followed up at 1, 6, and 12 months and annually after that, as previously described (20, 21). Medical records, phone calls, and clinical visits were used to collect follow-up data by well-trained cardiologists unaware of the study’s goal. If ischemia episodes were suspected, patients were advised to return for coronary angiography. The median follow-up period was 881 days [interquartile range (IQR): 807–944 days].

### Definitions and Clinical Outcomes

Based on the Fourth Universal Definition of AMI, AMI was defined as a rise in cardiac biomarkers above the 99th percentile of the normal upper limit, in conjunction with symptoms of ischemia, electrocardiographic changes, or abnormal imaging findings (22). MACE was a composite of death from any cause, AMI, and revascularization.

### Biochemical Analysis

Standard hospital assays measured TG, TC, and HDL-C (Determiner L HDL; Kyowa Medex, Tokyo, Japan) in fasting status. Lp(a) was determined by the immunoturbidimetry method [LASAY Lp(a) auto; SHIMA Laboratories Co., Ltd.]. dLDL-C was measured using the selective solubilization method (low-density lipid cholesterol test kit; Kyowa Medex, Tokyo, Japan). Estimated LDL-C levels were calculated using the Martin/Hopkins, Sampson, and Friedewald equations. The ratio between triglycerides and VLDL-C depends on the TG and non-HDL-C levels. To convert TC, HDL-C, and LDL-C values from milligrams per deciliter to millimoles per liter, multiply by 0.0259. To convert TG values from milligrams per deciliter to millimoles per liter, multiply by 0.0113.

### Other Covariates

Body mass index (BMI) was calculated by dividing body weight (in kilogram) by height in meters squared. The estimated glomerular filtration rate (eGFR) was calculated according to the MDRD GFR equation. Diabetes was diagnosed by fasting plasma glucose ≥7.0 mmol/L, the 2-h plasma glucose of the oral glucose tolerance test ≥11.1 mmol/L, those with hemoglobin A1c (HbA1c) ≥6.5% at baseline, or current use of hypoglycemic drugs or insulin (23). Hypertension was defined as self-reported hypertension, currently taking antihypertensive drugs, or recorded systolic blood pressure ≥140 mmHg or diastolic blood pressure ≥90 mmHg three or more consecutive times. Peripheral artery disease (PAD) was defined as a history of surgical or percutaneous peripheral artery revascularization or a stenosis ≥50% at Doppler ultrasound imaging in a peripheral artery district (extracranial carotids or lower limbs). Complete revascularization means all stenotic vessels greater than a defined diameter are revascularized, or all stenotic main-branch vessels are revascularized.

### Statistical Analysis

The association between dLDL-C and calculated LDL-C was plotted as a heated scatterplot with a linear regression line. All continuous values did not fit the normal distribution and were all reported as median and IQR (25th–75th percentiles). Categorical variables were described using frequencies and percentages.

Correct estimation was defined as estimated LDL-C in the same category as dLDL-C based on the following dLDL-C levels: less than 1.4, 1.4–1.8, 1.8–2.6, 2.6–3.0, and 3.0 mmol/L or greater. Misclassification, including both overestimation and underestimation, was assessed for each LDL-C estimation method compared with dLDL-C measurement. The underestimation group was defined as the underestimation of the LDL-C category by the equation method compared to the direct measurement. Similarly, the overestimated group was defined as the overestimation of the LDL-C category by the equation method compared to the direct measurement. Misclassification on the risk of adverse outcomes between dLDL-C and calculated LDL-C was investigated by Cox regression models with patients divided into three groups. The Schoenfeld Residuals Test is used to test the proportional hazard assumption in Cox model. The distributions of dLDL-C, TG, TC, lipoprotein(a), HDL-C, and non-HDL-C were graphed as density plots using the same three groups.

Univariate and multivariate Cox regression models estimated hazard ratios for all-cause mortality, recurrent AMI, and MACE. Multivariate analyses were adjusted for clinically associated variables with prognosis, i.e., age, sex, current smoker, eGFR <60 ml/min, complete revascularization, BMI, TG, Lp(a), statin, prior AMI, prior PCI, prior CABG, prior stroke, ejection fraction, hypertension, and diabetes mellitus. The Kaplan–Meier approach was used to calculate the cumulative incidence of clinical outcomes. The log-rank test was used to compare Kaplan–Meier survival curves. LDL-C was also estimated by the Friedewald and Sampson equation for sensitivity analysis.

## Results

### Baseline Characteristics

The baseline characteristics of the 9,751 individuals are reported in Table 1. Median age was 59.00 years, 7,512 (76.7%) patients were men, and 56.8% of patients were current smokers, 1,111 (11.4%) patients were identified as having ST-segment elevation myocardial infarction (STEMI), 1,007 (10.3%) patients were diagnosed with non-STEMI, 4,416 (45.3%) subjects were diagnosed with diabetes, 6,304 (64.6%) patients suffered from hypertension, 6,565 (67.3%) subjects were diagnosed with hyperlipemia. A total of 123 (1.3%) all-cause death, 114 (1.2%) recurrent AMI, and 1,035 (10.6%) MACE were recorded during the follow-up. All participants were separated into five groups based on classification levels for directly measured and calculated LDL-C by the Martin Equation. We found statistically significant differences among the five groups in ejection fraction, STEMI, non-STEMI, lipoprotein(a), TC, TG, HDL-C, non-HDL-C, LDL-C, and hyperlipidemia. Compared to the correct estimation group, the 1 category over group had a higher proportion of STEMI (14.7% vs. 11.4%), higher median TG (1.86 vs. 1.53), and non-HDL-C levels (3.13 vs. 2.99) but lower levels of dLDL-C (1.79 vs. 2.33), and lipoprotein(a) (164.27 vs. 189.73) values. The baseline characteristics of the five groups based on classification levels for dLDL-C and calculated LDL-C by the Friedewald equation and Sampson equation are illustrated in Supplementary Tables 1, 2, respectively.

**TABLE 1:** Baseline features and adverse outcomes according to concordance between the Martin equation and direct method.

	Overall	Underestimated		Correct estimation	Overestimated		*P*
		≥2 categories under	1 category under		1 category over	≥2 categories over	
*n*	9,751	20	924	8,010	768	29	
Age, years	59.00 (51.00, 66.00)	57.50 (52.00, 62.75)	59.00 (53.00, 66.00)	59.00 (51.00, 66.00)	58.00 (50.00, 65.00)	55.00 (51.00, 59.00)	0.086
Male	7,512 (77.0)	15 (75.0)	707 (76.5)	6,188 (77.3)	581 (75.7)	21 (72.4)	0.811
Current smoker	5,536 (56.8)	10 (50.0)	497 (53.8)	4,555 (56.9)	458 (59.6)	16 (55.2)	0.174
BMI	25.90 (23.88, 27.76)	24.98 (22.55, 27.43)	25.82 (24.02, 27.77)	25.91 (23.88, 27.76)	25.91 (23.87, 27.76)	25.71 (24.68, 27.04)	0.807
eGFR <60 mL/min	470 (4.8)	1 (5.0)	36 (3.9)	388 (4.8)	45 (5.9)	0 (0.0)	0.287
Ejection fraction	63.60 (60.00, 67.10)	64.50 (59.75, 68.40)	64.00 (60.10, 68.00)	63.40 (60.00, 67.00)	63.00 (60.00, 67.00)	63.00 (60.00, 65.00)	0.003
STEMI	1,111 (11.4)	0 (0.0)	82 (8.9)	911 (11.4)	113 (14.7)	5 (17.2)	0.001
Non-STEMI	1,007 (10.3)	2 (10.0)	68 (7.4)	848 (10.6)	84 (10.9)	5 (17.2)	0.025
**Lipid values**							
Lipoprotein(a), mg/L	185.38 (77.90, 411.88)	124.46 (76.21, 238.69)	170.40 (63.78, 382.71)	189.73 (81.02, 419.03)	164.27 (67.97, 389.62)	88.82 (58.20, 253.63)	<0.001
TC, mmol/L	4.03 (3.43, 4.78)	4.27 (3.47, 4.51)	4.00 (3.23, 4.49)	4.04 (3.47, 4.90)	4.00 (3.30, 4.61)	5.33 (4.49, 5.76)	<0.001
TG, mmol/L	1.53 (1.14, 2.10)	1.19 (0.86, 1.86)	1.34 (1.02, 1.76)	1.53 (1.15, 2.08)	1.86 (1.31, 2.90)	5.70 (5.00, 6.98)	<0.001
HDL-C, mmol/L	0.99 (0.84, 1.17)	1.25 (1.00, 1.63)	1.08 (0.92, 1.29)	0.99 (0.84, 1.17)	0.88 (0.76, 1.04)	0.80 (0.75, 0.95)	<0.001
Non-HDL-C, mmol/L	2.99 (2.42, 3.74)	2.84 (2.06, 3.05)	2.95 (2.14, 3.37)	2.99 (2.47, 3.83)	3.13 (2.37, 3.69)	4.57 (3.71, 4.96)	<0.001
Direct LDL-C, mmol/L	2.33 (1.85, 2.99)	3.05 (2.55, 3.08)	2.61 (1.83, 2.98)	2.33 (1.92, 3.11)	1.79 (1.62, 2.57)	2.13 (1.22, 2.46)	<0.001
Friedewald LDL-C, mmol/L	2.19 (1.71, 2.84)	1.75 (1.26, 2.40)	2.22 (1.54, 2.57)	2.21 (1.77, 2.97)	1.86 (1.49, 2.59)	1.96 (0.87, 2.17)	<0.001
Martin/Hopkins LDL-C, mmol/L	2.34 (1.86, 2.97)	2.13 (1.55, 2.41)	2.40 (1.66, 2.61)	2.34 (1.92, 3.11)	2.13 (1.83, 2.79)	3.03 (1.99, 3.10)	<0.001
Sampson LDL-C, mmol/L	2.29 (1.80, 2.93)	2.02 (1.36, 2.45)	2.35 (1.62, 2.62)	2.29 (1.87, 3.07)	1.95 (1.71, 2.69)	2.45 (1.64, 2.58)	<0.001
**Past medical history**							
Diabetes mellitus	4,416 (45.3)	7 (35.0)	397 (43.0)	3,641 (45.5)	358 (46.6)	13 (44.8)	0.477
Hypertension	6,304 (64.6)	11 (55.0)	593 (64.2)	5,176 (64.6)	504 (65.6)	20 (69.0)	0.833
Hyperlipidemia	6,565 (67.3)	13 (65.0)	573 (62.0)	5,417 (67.6)	537 (69.9)	25 (86.2)	0.001
Previous myocardial infarction	1,886 (19.3)	5 (25.0)	153 (16.6)	1,586 (19.8)	134 (17.4)	8 (27.6)	0.058
Previous percutaneous coronary intervention	2,302 (23.6)	5 (25.0)	205 (22.2)	1,899 (23.7)	185 (24.1)	8 (27.6)	0.835
Previous coronary artery bypass graft	405 (4.2)	0 (0.0)	35 (3.8)	333 (4.2)	36 (4.7)	1 (3.4)	0.779
Previous stroke or transient ischemic attack	1,029 (10.6)	2 (10.0)	105 (11.4)	822 (10.3)	97 (12.6)	3 (10.3)	0.3
Peripheral artery disease	727 (7.5)	1 (5.0)	74 (8.0)	593 (7.4)	59 (7.7)	0 (0.0)	0.556
**Procedural characteristics**							
Multivessel disease	7,357 (75.4)	14 (70.0)	686 (74.2)	6,040 (75.4)	593 (77.2)	24 (82.8)	0.528
Percutaneous coronary intervention with DES	476 (4.9)	2 (10.0)	47 (5.1)	387 (4.8)	38 (4.9)	2 (6.9)	0.824
Complete revascularization	5,238 (53.7)	14 (70.0)	469 (50.8)	4,332 (54.1)	409 (53.3)	14 (48.3)	0.183
**Medical therapy at discharge**							
Aspirin	9,637 (98.8)	20 (100.0)	915 (99.0)	7,916 (98.8)	758 (98.7)	28 (96.6)	0.742
Clopidogrel	9,610 (98.6)	20 (100.0)	903 (97.7)	7,902 (98.7)	756 (98.4)	29 (100.0)	0.218
Statin	9,367 (96.1)	20 (100.0)	888 (96.1)	7,698 (96.1)	732 (95.3)	29 (100.0)	0.526
β Blockers	8,805 (90.3)	17 (85.0)	834 (90.3)	7,222 (90.2)	707 (92.1)	25 (86.2)	0.396
ACE inhibitors or ARBs	5,434 (55.7)	15 (75.0)	500 (54.1)	4,486 (56.0)	419 (54.6)	14 (48.3)	0.256
Oral anticoagulation	40 (0.4)	0 (0.0)	2 (0.2)	35 (0.4)	3 (0.4)	0 (0.0)	0.878
**Study outcomes**							
All-cause mortality	123 (1.3)	0 (0.0)	14 (1.5)	100 (1.2)	9 (1.2)	0 (0.0)	0.884
Recurrent acute myocardial infarction	114 (1.2)	0 (0.0)	13 (1.4)	95 (1.2)	5 (0.7)	1 (3.4)	0.434
MACE	1,035 (10.6)	3 (15.0)	95 (10.3)	847 (10.6)	84 (10.9)	6 (20.7)	0.446

*Data are n/N (%) or median (IQR). BMI, body mass index; eGFR, estimated glomerular filtration rate; STEMI, ST-segment elevation myocardial infarction; TC, total cholesterol; TG, triglyceride; HDL-C, high-density lipoprotein cholesterol; LDL-C, low-density lipoprotein cholesterol DES, drug eluting stent; ACE, angiotensin-converting enzyme; ARBs, angiotensin-II receptor blockers; MACE, major adverse cardiovascular event.*

### Correlation of Low-Density Lipoprotein Cholesterol Values Among Equations and Direct Measurement

**FIGURE 1:**
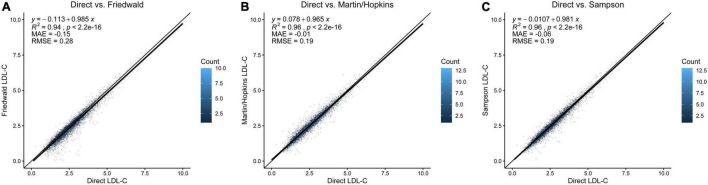
Comparison of direct LDL-C and LDL-C equations. **(A)** Direct measurement vs. Friedewald equation. **(B)** Direct measurement vs. Martin/Hopkins equation. **(C)** Direct measurement vs. Sampson equation. The diagonal line is the unity line in each graph, where both equations estimate the same value. Each dot represents the estimated LDL-C by the respective equation indicated on the *x*- and *y*-axes. The dot’s color represents data density from light blue to dark blue. LDL-C, low-density lipoprotein cholesterol.

The Friedewald equation produced lower LDL-C in most patients than the direct method (Figure 1A). Compared to the Friedewald equation (Figure 1A, *R*^2^ = 0.94, MAE = −0.15 mmol/L, RMSE = 0.28 mmol/L), the Martin/Hopkins (Figure 1B, *R*^2^ = 0.96, MAE = −0.01 mmol/L, RMSE = 0.19 mmol/L), and Sampson (Figure 1C, *R*^2^ = 0.96, MAE = −0.06, RMSE = 0.19 mmol/L) equations had a stronger correlation with dLDL-C at all levels.

### The Misclassification Proportion Referenced by Direct Measurement at Different Low-Density Lipoprotein Cholesterol Levels

**FIGURE 2:**
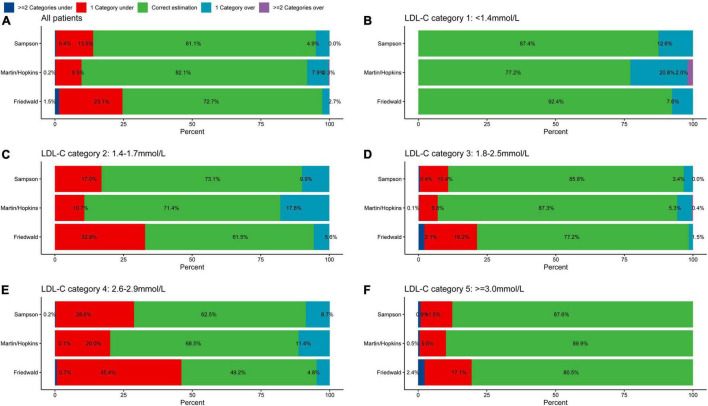
Proportion of misclassified patients per direction by estimated LDL-C category. Graphs represent the total percentage of under classified and overclassified patients within each LDL-C category. Values to the left and right of 0 on the *x*-axis indicate percentage under classified and percentage overclassified, respectively. Proportion of misclassification in **(A)** all patients, **(B)** LDL-C category 1: <1.4 mmol/L, **(C)** LDL-C category 2: 1.4–1.7 mmol/L, **(D)** LDL-C category 3: 1.8–2.5 mmol/L, **(E)** LDL-C category 4: 2.6–2.9 mmol/L, and **(F)** LDL-C category 5: ≥3.0 mmol/L. LDL-C, low-density lipoprotein cholesterol.

In the whole population, underestimation of LDL-C was found in 9.7% of patients using the Martin/Hopkins equation, 13.9% using the Sampson equation, and 24.6% using the Friedewald equation referenced by direct measurement (Figure 2A). The proportion of overestimated LDL-C classification was relatively low, 2.7% (Friedewald), 4.9% (Sampson), and 8.2% (Martin/Hopkins), respectively. The Martin/Hopkins equation had the highest percentage of correct categorization, at 82.1%, compared to 81.1% for the Sampson equation and 72.7% for the Friedewald equation. In the direct measurement of LDL-C <1.4 mmol/L (Figure 2B), each equation has a good consistency with the direct measurement method. In each classification with LDL-C greater than 1.4 mmol/L, the equation method significantly underestimated the LDL-C classification. Underestimation of LDL-C values of 1.4–1.7 mmol/L occurred in 10.7% of cases using the Martin/Hopkins equation, compared to 17.0% using the Sampson equation and 32.9% using the Friedewald equation (Figure 2C). In conclusion, the Martin/Hopkins equation had the least under classification in each LDL-C category compared to the Friedewald and Sampson equation (Figures 2A–F).

### Association Between Direct Low-Density Lipoprotein Cholesterol and Calculated Low-Density Lipoprotein Cholesterol With Risk of All-Cause Mortality, Recurrent Acute Myocardial Infarction, and Major Adverse Cardiovascular Event

**FIGURE 3:**
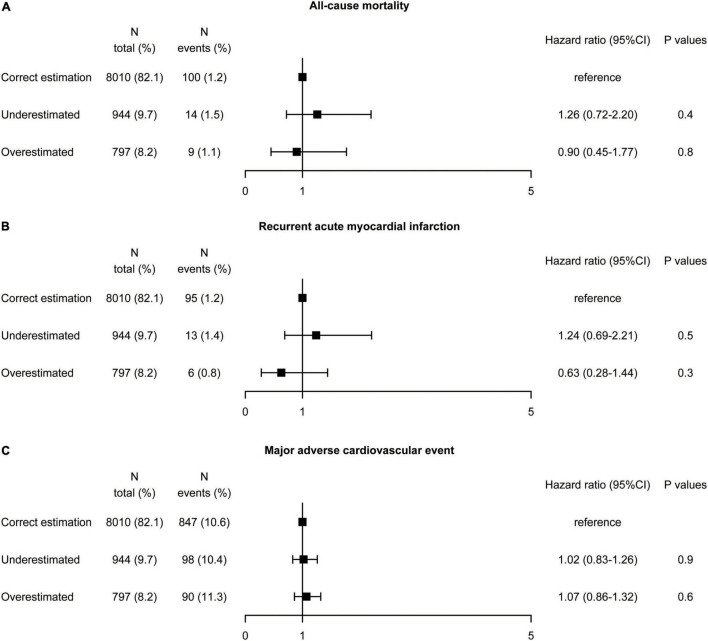
Association between misclassification by the Martin equation and the risk of **(A)** all-cause mortality, **(B)** recurrent acute myocardial infarction, and **(C)** major adverse cardiovascular event by univariate analysis. Hazard ratios were from Cox proportional hazards regressions with univariable analysis. CI, confidence interval.

Discordance in the risk of adverse events between dLDL-C and calculated LDL-C by the Martin equation was examined (Figure 3). Patients were divided into three according to (i) correct estimation group: calculated LDL-C category in the same category as dLDL-C (reference), (ii) underestimated group: underestimation of the LDL-C category by the equation compared to the dLDL-C (iii) overestimated group: overestimation of the LDL-C category by the equation compared to the dLDL-C. As depicted in Supplementary Figure 1, the Schoenfeld Residuals Test demonstrated that proportional hazards assumption was met (all *P* for global Schoenfeld test >0.05). Univariate Cox regression analysis showed that compared the correct estimation group, the underestimated group did not reduce all-cause mortality (HR 1.26, 95% CI: 0.72–2.20, *P* = 0.4), recurrent AMI (HR 1.24, 95% CI: 0.69–2.21, *P* = 0.5), and MACE (HR 1.02, 95% CI: 0.83–1.26, *P* = 0.9). Similarly, the overestimated group did not exacerbate all-cause mortality (HR 0.9, 95% CI: 0.45–1.77, *P* = 0.8), recurrent AMI (HR 0.63, 95% CI: 0.28–1.44, *P* = 0.3), and MACE (HR 1.07, 95% CI: 0.86–1.32, *P* = 0.6). We performed a sensitivity analysis with different triglycerides levels [<1.7 and ≥1.7 mmol/L (150 mg/dL)] due to the lower triglycerides in Asian population. The underestimated and overestimated groups were not associated with a higher or lower risk of all-cause mortality, recurrent AMI, and MACE compared with the correct estimation group according to different triglycerides levels (Supplementary Figures 2, 3).

**FIGURE 4:**
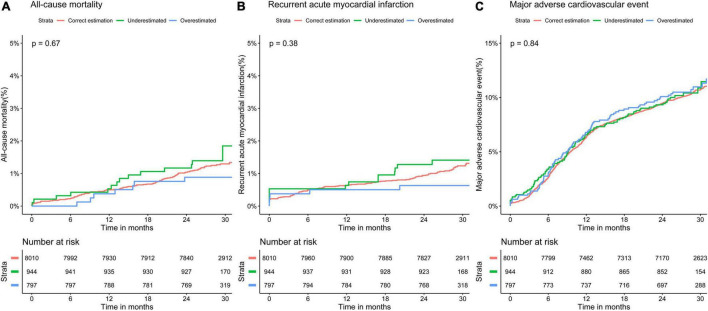
Kaplan–Meier curves for 30-month adverse event rates in different classifications of low-density lipoprotein cholesterol by the Martin equation. Overall rates of **(A)** all-cause mortality, **(B)** recurrent acute myocardial infarction, and **(C)** major adverse cardiovascular event.

K-M survival analyses (Figure 4) indicated no significant differences in all-cause mortality, recurrent AMI, and MACE between the three groups (all-cause mortality: log-rank *P* = 0.67; recurrent AMI: log-rank *P* = 0.38; MACE: log-rank *P* = 0.84).

When multivariate-adjusted hazard ratios were further adjusted for age, sex, current smoker, eGFR <60 ml/min, complete revascularization, BMI, TG, Lp(a), statin, prior AMI, prior PCI, prior CABG, prior stroke, ejection fraction, hypertension, and diabetes mellitus, the underestimated and overestimated groups were not associated with a higher or lower risk of all-cause mortality, recurrent AMI, and MACE compared with the correct estimation group (Supplementary Figure 4).

Univariate, K-M survival and multivariate analyses results were robust when LDL-C was estimated by the Friedewald (Supplementary Figures 5–7) and Sampson equation (Supplementary Figures 8–10) instead of the Martin/Hopkins equation.

### Distributions of Lipid Values Between Three Groups

**FIGURE 5:**
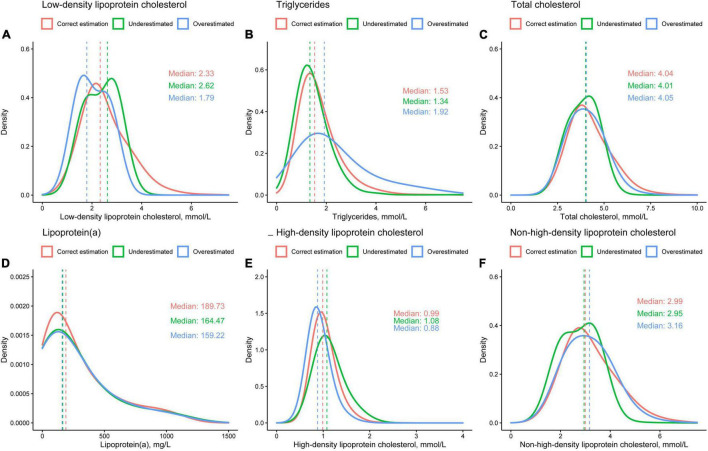
Distributions of **(A)** directly measured low-density lipoprotein cholesterol, **(B)** triglycerides, **(C)** total cholesterol, **(D)** lipoprotein(a), **(E)** high-density lipoprotein cholesterol, and **(F)** non-high-density lipoprotein cholesterol.

To investigate possible reasons for the fact that there is no difference in risk of adverse events between the three groups, concentration distributions of dLDL-C (Figure 5A), TG (Figure 5B), TC (Figure 5C), lipoprotein(a) (Figure 5D), HDL-C (Figure 5E), and non-HDL-C (Figure 5F) for the three groups were plotted. In the correct estimation, underestimated and overestimated group, the median dLDL-C was 2.33, 2.62, and 1.79 mmol/L, the median TG was 1.53, 1.34, and 1.92 mmol/L, the median TC was 4.04, 4.01, and 4.05 mmol/L, the median lipoprotein(a) were 189.73, 164.47, and 159.22 mg/L, the median HDL-C were 0.99, 1.08, and 0.88 mmol/L, the median non-HDL-C was 2.99, 2.95, and 3.16 mmol/L, respectively. This illustrates that differences in LDL-C and TG were significant between the three groups, while differences in other lipid items, including TC, lipoprotein(a), HDL-C, and non-HDL-C, were slight.

### Subgroup Analysis in Patients With Diabetes

**FIGURE 6:**
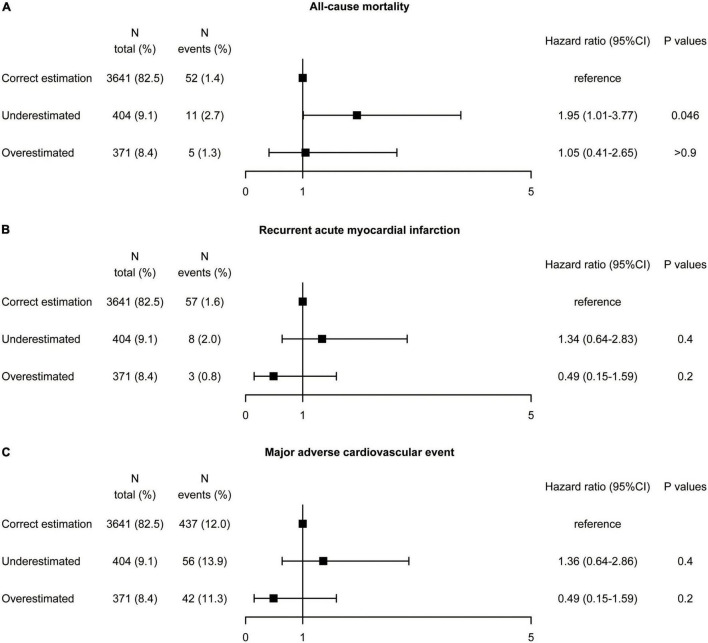
Association between misclassification by the Martin equation and the risk of **(A)** all-cause mortality, **(B)** recurrent acute myocardial infarction, and **(C)** major adverse cardiovascular event by multivariate analysis in patients with diabetes. Hazard ratios were from Cox proportional hazards regressions with multivariable analysis. CI, confidence interval.

The results of the diabetes subgroup analysis were similar to those of the whole population. In the diabetes population, underestimation of LDL-C was found in 9.2% of patients using the Martin/Hopkins equation, 4.4% using the Sampson equation, and 25.6% using the Friedewald equation referenced by direct measurement (Supplementary Figure 11). The proportion of overestimated LDL-C classification was relatively low, 2.5% (Friedewald), 4.8% (Sampson), and 8.4% (Martin/Hopkins), respectively. Multivariate Cox regression analysis in diabetes patients (Figure 6) showed that compared the correct estimation group, the underestimated group did not reduce recurrent AMI (HR 1.34, 95% CI: 0.64–2.83, *P* = 0.4), and MACE (HR 1.36, 95% CI: 0.64–2.86, *P* = 0.4). The all-cause mortality rate seemed to be higher in the underestimated LDL-C category (HR 1.95, 95% CI: 1.01–3.77, *P* = 0.046), however the *P* for interaction was 0.063. The overestimated group did not exacerbate all-cause mortality (HR 1.05, 95% CI: 0.41–2.65, *P* = 0.8), recurrent AMI (HR 0.49, 95% CI: 0.15–1.59, *P* = 0.2), and MACE (HR 1.07, 95% CI: 0.86–1.32, *P* = 0.6).

## Discussion

In our real-world cohort of 9,751 participants from the Fuwai PCI registry, this is the first study to examine whether dLDL-C measurement identifies additional patients at increased risk of adverse events compared with estimated LDL-C by equations in patients with CAD in an Asian population. There were several significant findings: (1) compared with the dLDL-C measurement, patients who were underclassified and overclassified using the Martin/Hopkins equation were present in 17.9% of individuals in terms of LDL-C classification, which was slightly lower than the Sampson (18.9%) and Friedewald (27.3%) equations; (2) misclassification (underestimation and overestimation) of LDL-C category by all three estimated LDL-C equations compared with dLDL-C was not associated with a higher or lower risk of all-cause death, recurrent AMI, and MACE; (3) there was also a discordance between dLDL-C and calculated LDL-C in diabetes, but it was not associated with prognosis. Furthermore, the estimated LDL-C by the Martin/Hopkins and Sampson equations is cost-effective and may be an alternative option for LDL-C measurement.

Low-density lipoprotein cholesterol can be measured directly or *via* predictive models in clinical laboratories. Because it is unaffected by chylomicrons or TG-rich lipoproteins, ultracentrifugation is primarily recognized as the most accurate method for measuring LDL-C. Due to the intense manual sample preparation and extended analysis time, the ultracentrifugation reference method for LDL-C detection is impracticable for modern routine laboratories. The clinical biochemistry laboratory faces a particular issue in directly detecting LDL-C. The Friedewald equation is used to estimate LDL-C in the majority of the published literature on cardiovascular disease prevention (11). However, it underestimates LDL-C when TG is near or more than 4.5 mmol/L (24, 25). In 2013, the Martin-Hopkins equation was created to calculate LDL-C more correctly and categorize people on lipid-lowering medications who had low LDL-C. External validation of Martin’s method has been conducted in multiple national and international datasets (8, 26–28), including those who received PCSK9i [Evolocumab (29) and Alirocumab (30)]. Li et al. discovered that the Sampson equation is more accurate for determining LDL-C in Chinese patients with acute coronary syndrome than the Friedewald equation (31). In 2020, Sampson et al. developed a new equation to estimate LDL-C with high reliability (14).

Direct LDL-C tests, which are commercially available, are homogeneous enzymatic colorimetric procedures that use cholesterol esterase and cholesterol oxidase to quantify lipids. To achieve selective solubilization of the non-LDL-C and LDL-C fractions, proprietary chemical detergents are used. The accuracy of dLDL-C measurement depends on the specificity of masking reagent. The bias of dLDL-C measurement increased in hypertriglyceridemia. Vesper found that the sample with the highest triglyceride value had the highest fail rate for LDL-C according to a laboratory community survey (32). Analogous to the Friedewald equation, the direct assay contains a portion of cholesterol in IDL and VLDL particles and may misestimate the true level of LDL-C (6). The accuracy of direct measurements is controversial, with different studies reaching different conclusions (7, 8, 33, 34). Mora et al. compared LDL-C values by Friedewald equation and direct method in relation to cardiovascular events in more than 20,000 healthy women. They discovered that the direct assay corresponded well with Friedewald calculations but that the direct assay was often lower, and the lower LDL-C concentrations by direct assay may misclassify a significant proportion of people into a lower risk category (34). Miller et al. compared eight direct measures of LDL-C with gold standard β quantification and found that in healthy people, three of the eight methods (Denka Seiken, Roche, and Sysmex International Reagents) met the requirements of the National Cholesterol Education Program (NCEP). However, for patients with dyslipidemia and cardiovascular diseases, none of the eight methods were satisfied (15). The study of Miida et al. showed opposite results, suggesting that Denka Seiken, Wako, Kyowa Medex, and Sekisui Medical could meet NCEP requirements in both healthy and patient populations, and their performance in non-fasting specimens was similar to that of fasting specimens (35). Friedewald equation underestimates LDL-C at low concentrations in people with diabetes (36, 37). Factors such as obesity, diabetes, and insulin resistance, which may affect variance in the TG: VLDL-C ratio, were not available for analysis in the Martin equation (13). In the subgroup analysis for patients with diabetes, all-cause mortality risk seemed to be higher in the underestimated LDL-C category. However, this trend was observed in subgroup analysis and was not consistent with the whole population. What is more, the *P*-value is very close to 0.05 (*P* = 0.046) and the interaction was not significant (*P* for interaction = 0.3). Therefore, the association between underestimated LDL-C category and the increased risk of all-cause mortality in patients with diabetes need more cautious to interpret the results. Further studies in diabetic patients are needed.

Our study’s strength is the substantial number of people who had both direct and computed LDL-C values, allowing for a head-to-head comparison. The goal of our research was to see if there were any changes in cardiovascular risk based on different LDL-C classification methods. In other words, it was to evaluate if dLDL-C vs. calculated LDL-C could identify a group of patients at increased risk of adverse events. This is important because there are differences between the Chinese guideline and the European and American guidelines in recommending LDL-C measurement methods. The Chinese guideline (17) recommends a direct measurement method. Due to the lack of a cost-effective, accurate, and widely available method to directly measure LDL-C, the evaluation of LDL-C in clinical research and clinical practice in European and American countries mainly relies on the equation method. The 2017 American College of Endocrinology guidelines for the management of dyslipidemia suggests that LDL-C can be estimated by the Friedewald equation but should be directly measured in patients with TG ≥2.8 mmol/L, diabetes or known vascular disease (Grade C; BEL 3) (19). The 2018 American Heart Association (AHA)/American College of Cardiology (ACC) guideline on the management of blood cholesterol stated that if calculated LDL-C <1.8 mmol/L by the Friedewald method, direct measurement or other equation is reasonable (IIa C-LD) (2). The 2019 European Society of Cardiology (ESC) guidelines for the management of dyslipidemias represented that the Friedewald equation is the most widely used clinical LDL-C measurement and that there are systemic errors and inaccuracies in the direct measurement of dyslipidemia (1). However, this guideline only stated the current situation of clinical application and did not recommend measurement methods. The 2021 lipid measurements in the management of cardiovascular diseases recommends that LDL-C can be estimated by TC, HDL-C, and TG measurements. For LDL-C ≥2.6 mmol/L and TG ≤1.7 mmol/L, the Friedewald formula is reasonable (IIa, B-NR). However, the Martin formula is recommended to estimate LDL-C in patients with TG <4.5 mmol/L (IIa, B-NR). For patients with TG level ≥4.5 mmol/L, equations to estimate LDL-C are not currently recommended (IIa, B-NR) (18).

### Limitations

This study is limited by several facets. First, this is an observational study from a large volume single-center cohort and may suffer from limited generalizability (38). Although the multivariate analysis was performed, we could not completely eliminate the influence of confounding factors. As a result, additional randomized clinical trials are needed to see if an intervention based on directly measured LDL-C is more effective than one based on estimated LDL-C in secondary prevention. Second, we did not evaluate trueness against β quantification, which is the reference measurement procedure of LDL-C. So, we cannot compare which of these approaches is closer to the gold standard. In addition, we did a *post hoc* analysis of outcome data collected prospectively from Fuwai PCI registry. This research is an exploratory study, and our results are hypothesis-generating.

## Conclusion

In conclusion, compared with dLDL-C measurement, misclassification (underestimation and overestimation) by the Martin/Hopkins and Sampson equations was present in approximately 20% of the patients. However, directly measured vs. calculated LDL-C did not identify any more individuals in the PCI population with increased risk of all-cause mortality, recurrent AMI, and MACE. Furthermore, the estimated LDL-C by the Martin/Hopkins and Sampson equations is cost-effective and may be an alternative option for LDL-C measurement, even in high-risk patients such as those with diabetes.

## Data Availability Statement

The raw data supporting the conclusions of this article will be made available by the authors, without undue reservation.

## Ethics Statement

The studies involving human participants were reviewed and approved by the Fuwai Hospital Ethics Committees. The patients/participants provided their written informed consent to participate in this study.

## Author Contributions

BS and H-YW: study design and interpretation of results. JL, LJ, ZC, and CS: data collection. BS, DY, and HW: data analysis. BS, H-YW, and K-FD: preparation of manuscript. BS, WS, and K-FD: revision of manuscript. All authors read and approved the final manuscript.

## Conflict of Interest

The authors declare that the research was conducted in the absence of any commercial or financial relationships that could be construed as a potential conflict of interest.

## Publisher’s Note

All claims expressed in this article are solely those of the authors and do not necessarily represent those of their affiliated organizations, or those of the publisher, the editors and the reviewers. Any product that may be evaluated in this article, or claim that may be made by its manufacturer, is not guaranteed or endorsed by the publisher.

## Abbreviations

TC: total cholesterol
LDL-C: low-density lipoprotein cholesterol concentration
CAD: coronary artery disease
TG: triglycerides
VLDL-C: very-low-density lipoprotein cholesterol
AMI: acute myocardial infarction
MACE: major adverse cardiovascular event
IQR: interquartile range
dLDL-C: direct LDL-C
BMI: body mass index
eGFR: estimated glomerular filtration rate
HbA1c: hemoglobin A1c
PAD: peripheral artery disease
STEMI: ST-segment elevation myocardial infarction
NCEP: National Cholesterol Education Program
AHA: American Heart Association
ACC: American College of Cardiology
ESC: European Society of Cardiology.
