# Supplementary material for: Directly Measured vs. Calculated Low-Density Lipoprotein Cholesterol Does Not Identify Additional Individuals With Coronary Artery Disease and Diabetes at Higher Risk of Adverse Events: Insight From a Large Percutaneous Coronary Intervention Cohort in Asia
Source: Front Cardiovasc Med. 2022 Jul 7;9:932878. doi: 10.3389/fcvm.2022.932878 (PMC9301080; doi:10.3389/fcvm.2022.932878)
Supplement: Supplementary file 1 [file Data_Sheet_1.DOCX]

**SUPPLEMENTAL MATERIALS**

**1. Supplemental tables**

**Supplemental Table 1: Baseline features and adverse outcomes according to concordance between the Friedewald equation and direct method**

|  | >=2 Categories under | 1 Category under | Concordant | 1 Category over | P |
| --- | --- | --- | --- | --- | --- |
| n | 146 | 2252 | 7093 | 260 |  |
| Age, years | 54.00 (48.00, 59.00) | 58.00 (50.00, 64.00) | 59.00 (51.00, 66.00) | 60.50 (53.00, 68.00) | <0.001 |
| Male | 124 (84.9) | 1735 (77.0) | 5455 (76.9) | 198 (76.2) | 0.149 |
| Current smoker | 93 (63.7) | 1284 (57.0) | 4009 (56.5) | 150 (57.7) | 0.364 |
| BMI | 26.70 (24.49, 28.69) | 26.06 (24.22, 28.09) | 25.80 (23.71, 27.72) | 25.18 (23.43, 27.04) | <0.001 |
| eGFR <60 mL/min | 3 (2.1) | 108 (4.8) | 348 (4.9) | 11 (4.2) | 0.432 |
| Ejection fraction | 65.00 (60.00, 68.40) | 64.00 (60.00, 67.12) | 63.40 (60.00, 67.10) | 63.00 (59.00, 66.00) | 0.024 |
| STEMI | 20 (13.7) | 230 (10.2) | 812 (11.4) | 49 (18.8) | <0.001 |
| non-STEMI | 14 (9.6) | 198 (8.8) | 759 (10.7) | 36 (13.8) | 0.016 |
| **Lipid values** |  |  |  |  |  |
| Lipoprotein (a), mg/L | 91.31 (34.96, 241.97) | 145.03 (59.47, 346.92) | 197.90 (85.95, 432.87) | 225.47 (101.45, 467.52) | <0.001 |
| TC, mmol/L | 4.51 (3.92, 5.22) | 3.99 (3.37, 4.59) | 4.04 (3.46, 4.93) | 3.78 (3.20, 4.51) | <0.001 |
| TG, mmol/L | 3.72 (2.25, 5.65) | 1.92 (1.39, 2.60) | 1.45 (1.10, 1.92) | 1.09 (0.86, 1.38) | <0.001 |
| HDL-C, mmol/L | 0.96 (0.79, 1.17) | 0.98 (0.84, 1.15) | 1.00 (0.84, 1.18) | 0.98 (0.80, 1.16) | 0.048 |
| Non-HDL-C, mmol/L | 3.55 (2.84, 4.24) | 2.99 (2.34, 3.56) | 2.99 (2.46, 3.86) | 2.58 (2.23, 3.50) | <0.001 |
| Direct LDL-C, mmol/L | 2.22 (1.93, 3.06) | 2.15 (1.82, 2.80) | 2.35 (1.91, 3.17) | 1.79 (1.65, 2.64) | <0.001 |
| Friedewald LDL-C, mmol/L | 1.39 (1.24, 2.38) | 1.78 (1.52, 2.54) | 2.23 (1.85, 3.08) | 2.03 (1.81, 3.00) | <0.001 |
| Martin/Hopkins LDL-C, mmol/L | 2.26 (1.82, 2.71) | 2.17 (1.75, 2.74) | 2.36 (1.95, 3.18) | 2.06 (1.81, 2.97) | <0.001 |
| Sampson LDL-C, mmol/L | 1.79 (1.54, 2.57) | 1.95 (1.68, 2.65) | 2.32 (1.92, 3.17) | 2.03 (1.82, 3.04) | <0.001 |
| **Past medical history** |  |  |  |  |  |
| Diabetes mellitus | 82 (56.2) | 1048 (46.5) | 3174 (44.7) | 112 (43.1) | 0.021 |
| Hypertension | 101 (69.2) | 1478 (65.6) | 4567 (64.4) | 158 (60.8) | 0.242 |
| Hyperlipidemia | 100 (68.5) | 1562 (69.4) | 4741 (66.8) | 162 (62.3) | 0.045 |
| Previous myocardial infarction | 28 (19.2) | 442 (19.6) | 1374 (19.4) | 42 (16.2) | 0.611 |
| Previous percutaneous coronary intervention | 32 (21.9) | 552 (24.5) | 1655 (23.3) | 63 (24.2) | 0.658 |
| Previous coronary artery bypass graft | 3 (2.1) | 111 (4.9) | 278 (3.9) | 13 (5.0) | 0.091 |
| Previous stroke or transient ischemic attack | 13 (8.9) | 227 (10.1) | 756 (10.7) | 33 (12.7) | 0.513 |
| Peripheral artery disease | 8 (5.5) | 175 (7.8) | 517 (7.3) | 27 (10.4) | 0.198 |
| **Procedural characteristics** |  |  |  |  |  |
| Multivessel disease | 110 (75.3) | 1702 (75.6) | 5349 (75.4) | 196 (75.4) | 0.999 |
| Percutaneous coronary intervention with DES | 4 (2.7) | 114 (5.1) | 346 (4.9) | 12 (4.6) | 0.65 |
| Complete revascularization | 84 (57.5) | 1190 (52.8) | 3848 (54.3) | 116 (44.6) | 0.012 |
| **Medical therapy at discharge** |  |  |  |  |  |
| Aspirin | 144 (98.6) | 2224 (98.8) | 7011 (98.8) | 258 (99.2) | 0.913 |
| Clopidogrel | 144 (98.6) | 2213 (98.3) | 6995 (98.6) | 258 (99.2) | 0.505 |
| Statin | 143 (97.9) | 2146 (95.3) | 6831 (96.3) | 247 (95.0) | 0.079 |
| β blockers | 135 (92.5) | 2060 (91.5) | 6376 (89.9) | 234 (90.0) | 0.127 |
| ACE inhibitors or ARBs | 80 (54.8) | 1220 (54.2) | 3990 (56.3) | 144 (55.4) | 0.383 |
| Oral anticoagulation | 0 (0.0) | 9 (0.4) | 31 (0.4) | 0 (0.0) | 0.614 |
| **Study outcomes** |  |  |  |  |  |
| All-cause mortality | 1 (0.7) | 26 (1.2) | 92 (1.3) | 4 (1.5) | 0.843 |
| Recurrent acute myocardial infarction | 3 (2.1) | 27 (1.2) | 83 (1.2) | 1 (0.4) | 0.495 |
| MACE | 16 (11.0) | 247 (11.0) | 737 (10.4) | 35 (13.5) | 0.406 |

Data are n/N (%) or median (IQR). BMI=body mass index. eGFR=estimated glomerular filtration rate. STEMI=ST-segment elevation myocardial infarction. TC = total cholesterol. TG = triglyceride. HDL-C=high-density lipoprotein cholesterol. LDL-C = low-density lipoprotein cholesterol DES=drug eluting stent. ACE=angiotensin-converting enzyme. ARBs=angiotensin-II receptor blockers. MACE=major adverse cardiovascular event.

**Supplemental Table 2: Baseline features and adverse outcomes according to concordance between the Sampson equation and direct method**

|  | >=2 Categories under | 1 Category under | Concordant | 1 Category over | >=2 Categories over | P |
| --- | --- | --- | --- | --- | --- | --- |
| n | 38 | 1319 | 7911 | 482 | 1 |  |
| Age, years | 55.00 (48.25, 59.00) | 58.00 (50.00, 64.00) | 59.00 (51.00, 66.00) | 59.00 (52.00, 67.00) | 53.00 (53.00, 53.00) | 0.005 |
| Male | 30 (78.9) | 1033 (78.3) | 6095 (77.0) | 353 (73.2) | 1 (100.0) | 0.237 |
| Current smoker | 22 (57.9) | 743 (56.3) | 4495 (56.8) | 275 (57.1) | 1 (100.0) | 0.923 |
| BMI | 26.33 (23.71, 28.01) | 26.08 (24.22, 28.07) | 25.86 (23.84, 27.76) | 25.35 (23.54, 27.34) | 24.68 (24.68, 24.68) | <0.001 |
| eGFR <60 mL/min | 1 (2.6) | 50 (3.8) | 391 (4.9) | 28 (5.8) | 0 (0.0) | 0.311 |
| Ejection fraction | 63.50 (60.00, 68.55) | 64.00 (60.00, 67.45) | 63.80 (60.00, 67.20) | 63.00 (60.00, 66.18) | 62.80 (62.80, 62.80) | 0.026 |
| STEMI | 4 (10.5) | 129 (9.8) | 893 (11.3) | 85 (17.6) | 0 (0.0) | <0.001 |
| non-STEMI | 4 (10.5) | 107 (8.1) | 828 (10.5) | 68 (14.1) | 0 (0.0) | 0.005 |
| **Lipid values** |  |  |  |  |  |  |
| Lipoprotein (a), mg/L | 108.76 (34.00, 350.05) | 141.28 (59.34, 336.70) | 190.89 (81.29, 421.77) | 217.34 (87.38, 446.93) | 26.58 (26.58, 26.58) | <0.001 |
| TC, mmol/L | 4.48 (3.78, 4.82) | 3.99 (3.28, 4.58) | 4.03 (3.46, 4.90) | 4.10 (3.30, 4.54) | 7.36 (7.36, 7.36) | <0.001 |
| TG, mmol/L | 2.66 (1.46, 6.67) | 1.71 (1.21, 2.46) | 1.52 (1.14, 2.06) | 1.34 (1.03, 1.83) | 7.74 (7.74, 7.74) | <0.001 |
| HDL-C, mmol/L | 1.04 (0.80, 1.34) | 1.02 (0.87, 1.20) | 0.99 (0.84, 1.17) | 0.95 (0.79, 1.12) | 0.84 (0.84, 0.84) | <0.001 |
| Non-HDL-C, mmol/L | 3.26 (2.45, 3.93) | 2.97 (2.22, 3.50) | 2.99 (2.46, 3.83) | 3.13 (2.34, 3.55) | 6.52 (6.52, 6.52) | <0.001 |
| Direct LDL-C, mmol/L | 3.00 (2.00, 3.07) | 2.60 (1.83, 2.82) | 2.33 (1.90, 3.11) | 2.29 (1.67, 2.59) | 2.56 (2.56, 2.56) | <0.001 |
| Friedewald LDL-C, mmol/L | 1.54 (0.82, 2.17) | 1.94 (1.45, 2.49) | 2.21 (1.77, 2.98) | 2.45 (1.74, 2.85) | 2.97 (2.97, 2.97) | <0.001 |
| Martin/Hopkins LDL-C, mmol/L | 2.02 (1.50, 2.59) | 2.29 (1.70, 2.69) | 2.34 (1.93, 3.11) | 2.58 (1.86, 2.95) | 3.87 (3.87, 3.87) | <0.001 |
| Sampson LDL-C, mmol/L | 1.84 (1.35, 2.47) | 2.17 (1.63, 2.58) | 2.29 (1.87, 3.08) | 2.60 (1.82, 2.99) | 3.25 (3.25, 3.25) | <0.001 |
| **Past medical history** |  |  |  |  |  |  |
| Diabetes mellitus | 20 (52.6) | 616 (46.7) | 3569 (45.1) | 211 (43.8) | 0 (0.0) | 0.515 |
| Hypertension | 20 (52.6) | 863 (65.4) | 5117 (64.7) | 303 (62.9) | 1 (100.0) | 0.409 |
| Hyperlipidemia | 25 (65.8) | 880 (66.7) | 5341 (67.5) | 319 (66.2) | 0 (0.0) | 0.603 |
| Previous myocardial infarction | 8 (21.1) | 244 (18.5) | 1552 (19.6) | 82 (17.0) | 0 (0.0) | 0.562 |
| Previous percutaneous coronary intervention | 7 (18.4) | 311 (23.6) | 1870 (23.6) | 114 (23.7) | 0 (0.0) | 0.927 |
| Previous coronary artery bypass graft | 1 (2.6) | 63 (4.8) | 318 (4.0) | 23 (4.8) | 0 (0.0) | 0.668 |
| Previous stroke or transient ischemic attack | 4 (10.5) | 128 (9.7) | 838 (10.6) | 59 (12.2) | 0 (0.0) | 0.628 |
| Peripheral artery disease | 2 (5.3) | 94 (7.1) | 591 (7.5) | 40 (8.3) | 0 (0.0) | 0.902 |
| **Procedural characteristics** |  |  |  |  |  |  |
| Multivessel disease | 27 (71.1) | 988 (74.9) | 5974 (75.5) | 367 (76.1) | 1 (100.0) | 0.898 |
| Percutaneous coronary intervention with DES | 2 (5.3) | 66 (5.0) | 382 (4.8) | 25 (5.2) | 1 (100.0) | 0.001 |
| Complete revascularization | 24 (63.2) | 693 (52.5) | 4273 (54.0) | 248 (51.5) | 0 (0.0) | 0.339 |
| **Medical therapy at discharge** |  |  |  |  |  |  |
| Aspirin | 37 (97.4) | 1304 (98.9) | 7819 (98.8) | 476 (98.8) | 1 (100.0) | 0.945 |
| Clopidogrel | 38 (100.0) | 1293 (98.0) | 7805 (98.7) | 473 (98.1) | 1 (100.0) | 0.361 |
| Statin | 37 (97.4) | 1265 (95.9) | 7607 (96.2) | 457 (94.8) | 1 (100.0) | 0.649 |
| β blockers | 34 (89.5) | 1205 (91.4) | 7131 (90.1) | 434 (90.0) | 1 (100.0) | 0.72 |
| ACE inhibitors or ARBs | 28 (73.7) | 712 (54.0) | 4430 (56.0) | 263 (54.6) | 1 (100.0) | 0.096 |
| Oral anticoagulation | 0 (0.0) | 5 (0.4) | 34 (0.4) | 1 (0.2) | 0 (0.0) | 0.945 |
| **Study outcomes** |  |  |  |  |  |  |
| All-cause mortality | 1 (2.6) | 13 (1.0) | 105 (1.3) | 4 (0.8) | 0 (0.0) | 0.665 |
| Recurrent acute myocardial infarction | 1 (2.6) | 16 (1.2) | 92 (1.2) | 5 (1.0) | 0 (0.0) | 0.937 |
| MACE | 5 (13.2) | 138 (10.5) | 839 (10.6) | 53 (11.0) | 0 (0.0) | 0.975 |

Data are n/N (%) or median (IQR). BMI=body mass index. eGFR=estimated glomerular filtration rate. STEMI=ST-segment elevation myocardial infarction. TC = total cholesterol. TG = triglyceride. HDL-C=high-density lipoprotein cholesterol. LDL-C = low-density lipoprotein cholesterol DES=drug eluting stent. ACE=angiotensin-converting enzyme. ARBs=angiotensin-II receptor blockers. MACE=major adverse cardiovascular event.

.

**2. Supplemental figures**


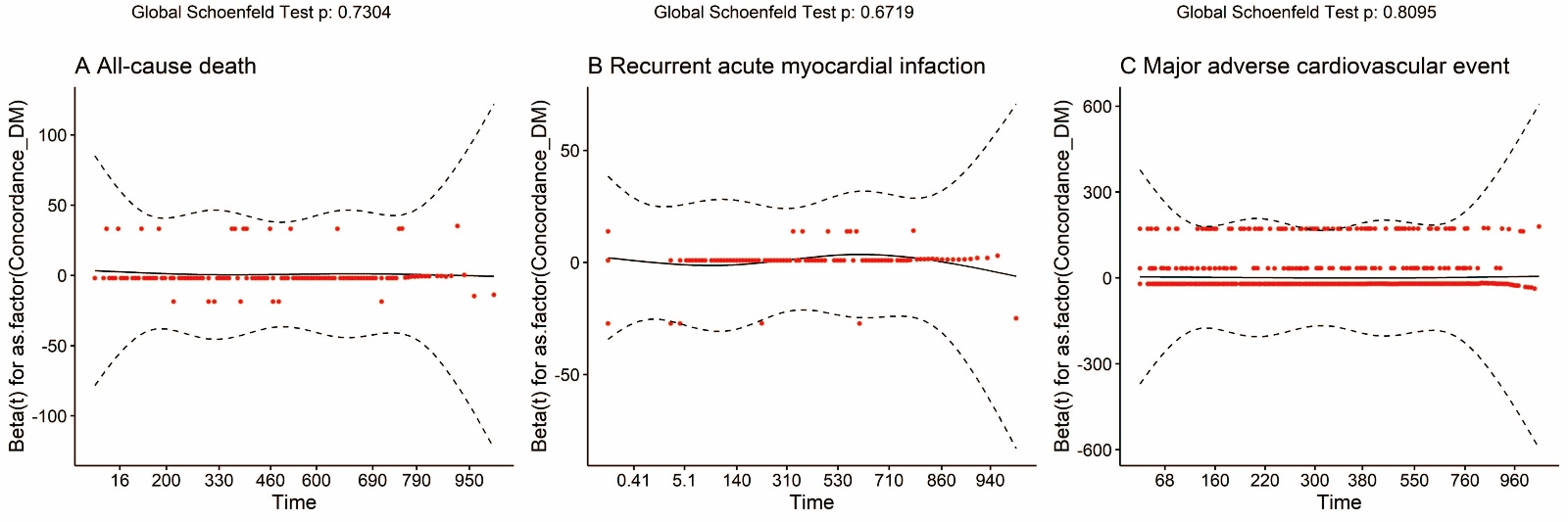


**Supplemental Figure 1 Schoenfeld Residuals Test for (A) all-cause mortality, (B) recurrent acute myocardial infarction, and (C) major adverse cardiovascular event.**

**
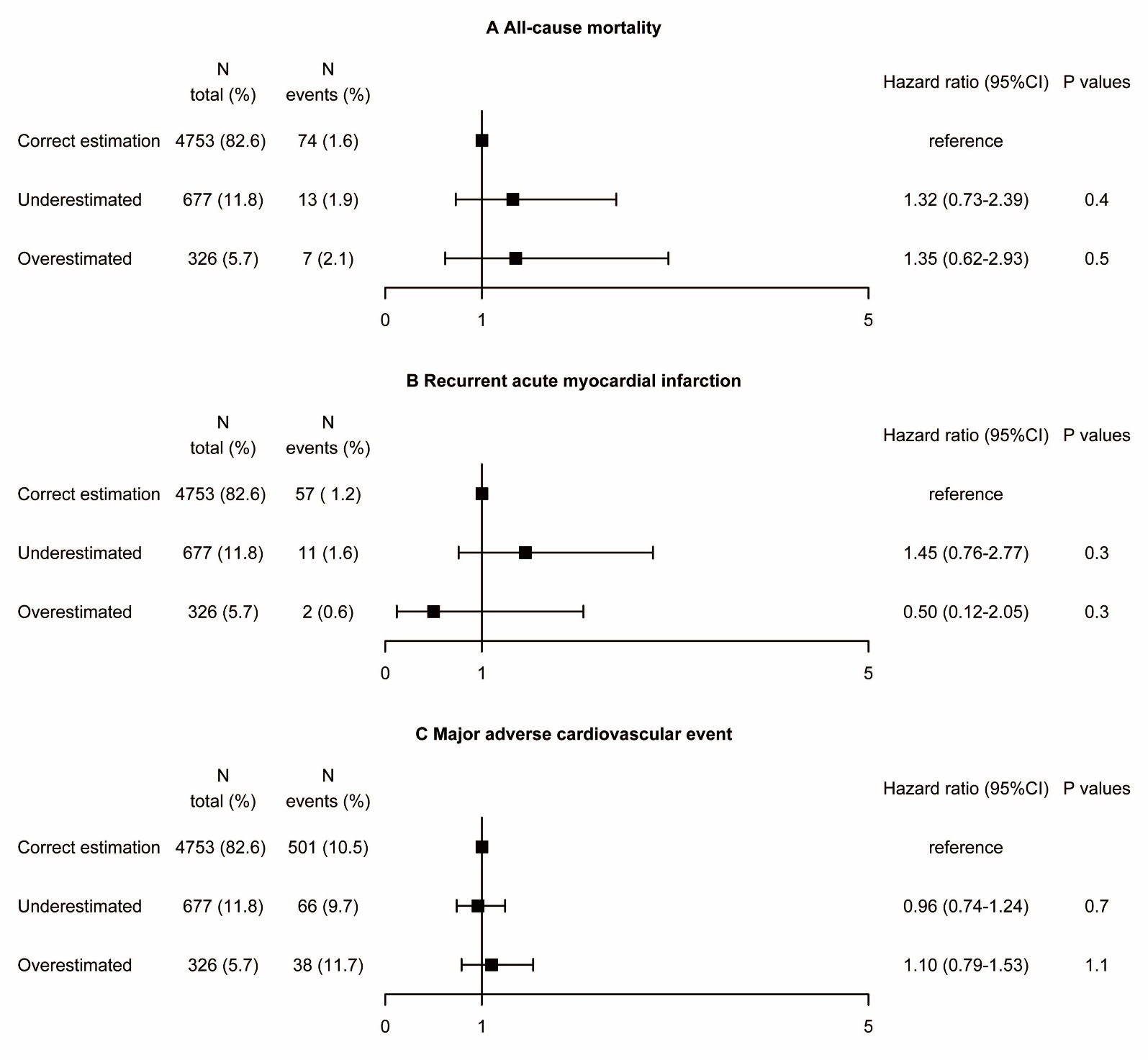
**

**Supplemental Figure 2 Association between misclassification by the Martin equation and the risk of (A) all-cause mortality, (B) recurrent acute myocardial infarction, and (C) major adverse cardiovascular event by univariate analysis in triglycerides <1.7 mmol/L (150 mg/dL).**

Hazard ratios were from Cox proportional hazards regressions with univariable analysis. CI = confidence interval.


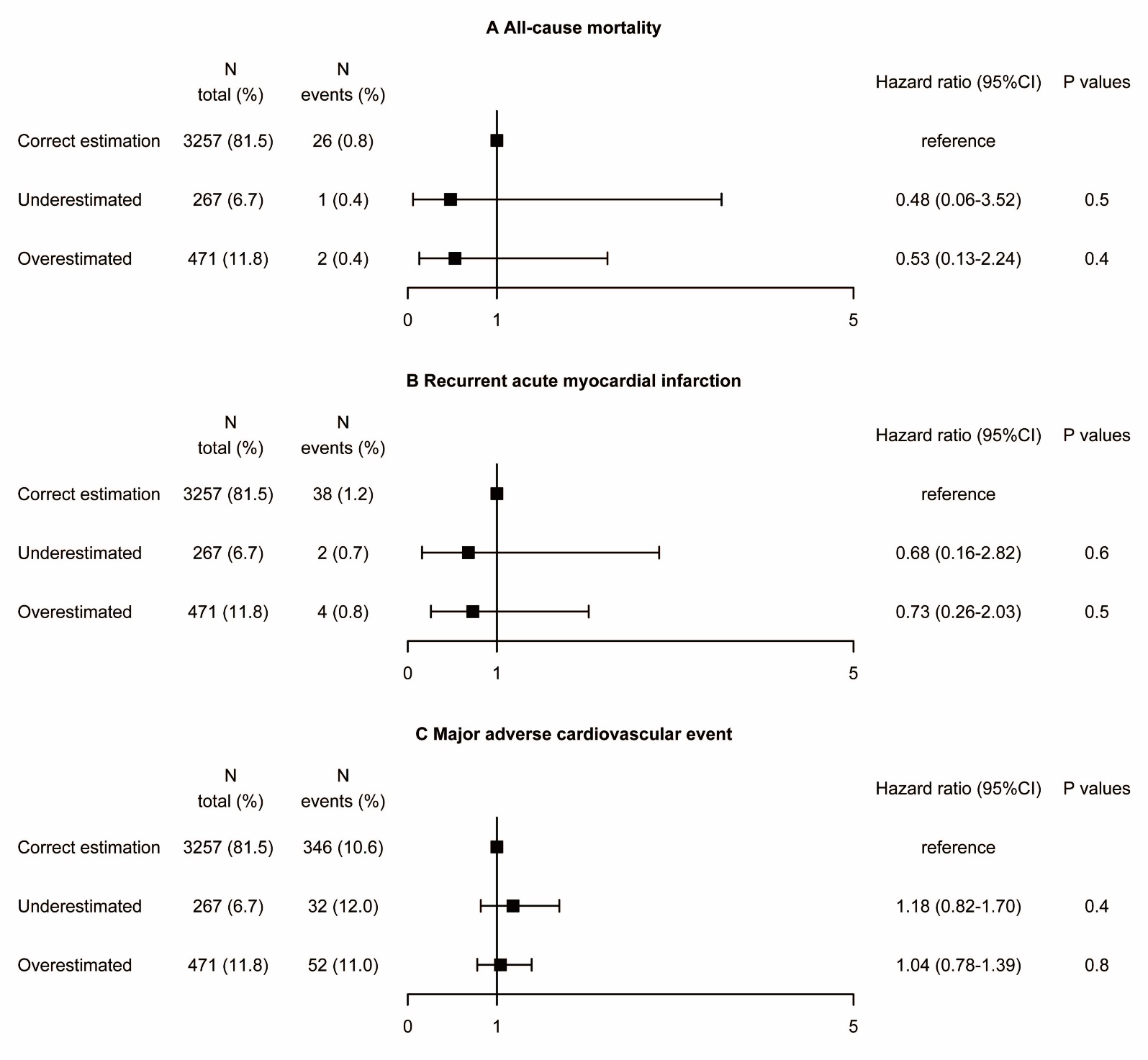
**Supplemental Figure 3 Association between misclassification by the Martin equation and the risk of (A) all-cause mortality, (B) recurrent acute myocardial infarction, and (C) major adverse cardiovascular event by univariate analysis in triglycerides >=1.7 mmol/L (150 mg/dL).**

Hazard ratios were from Cox proportional hazards regressions with univariable analysis. CI = confidence interval.

**
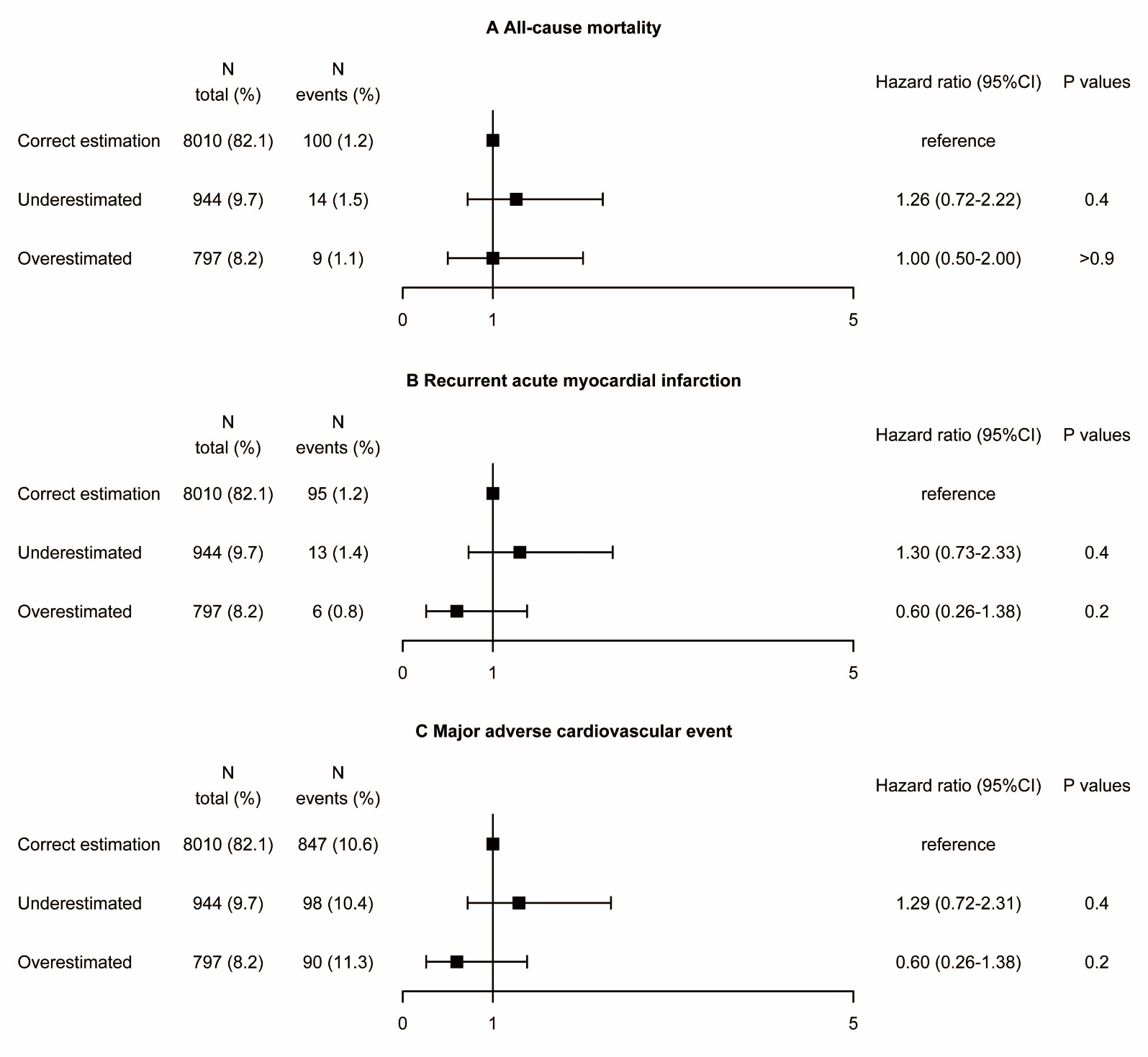
**

**Supplemental Figure 4 Association between misclassification by the Martin equation and the risk of (A) all-cause mortality, (B) recurrent acute myocardial infarction, and (C) major adverse cardiovascular event by multivariate analysis.**

Hazard ratios were from Cox proportional hazards regressions with multivariable adjustment for age, sex, current smoker, estimated glomerular filtration rate<60ml/min, complete revascularization, body mass index, ejection fraction, hypertension, and diabetes mellitus. CI = confidence interval.

**
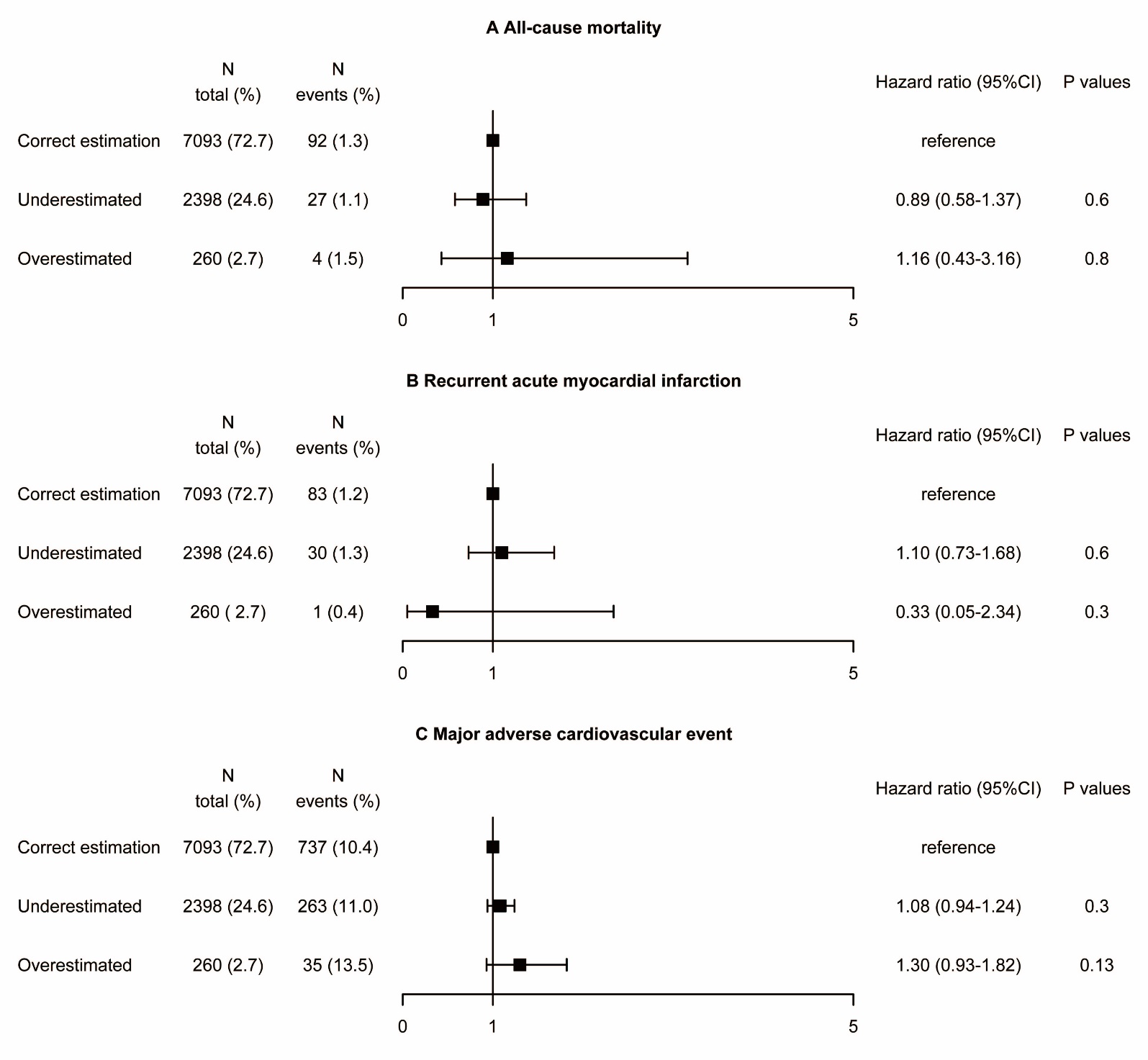
**

**Supplemental Figure 5 Association between misclassification by the Friedewald equation and the risk of** **(A) all-cause mortality, (B) recurrent acute myocardial infarction, and (C) major adverse cardiovascular event by univariate analysis.**

Hazard ratios were from Cox proportional hazards regressions with univariable analysis. CI = confidence interval.

**
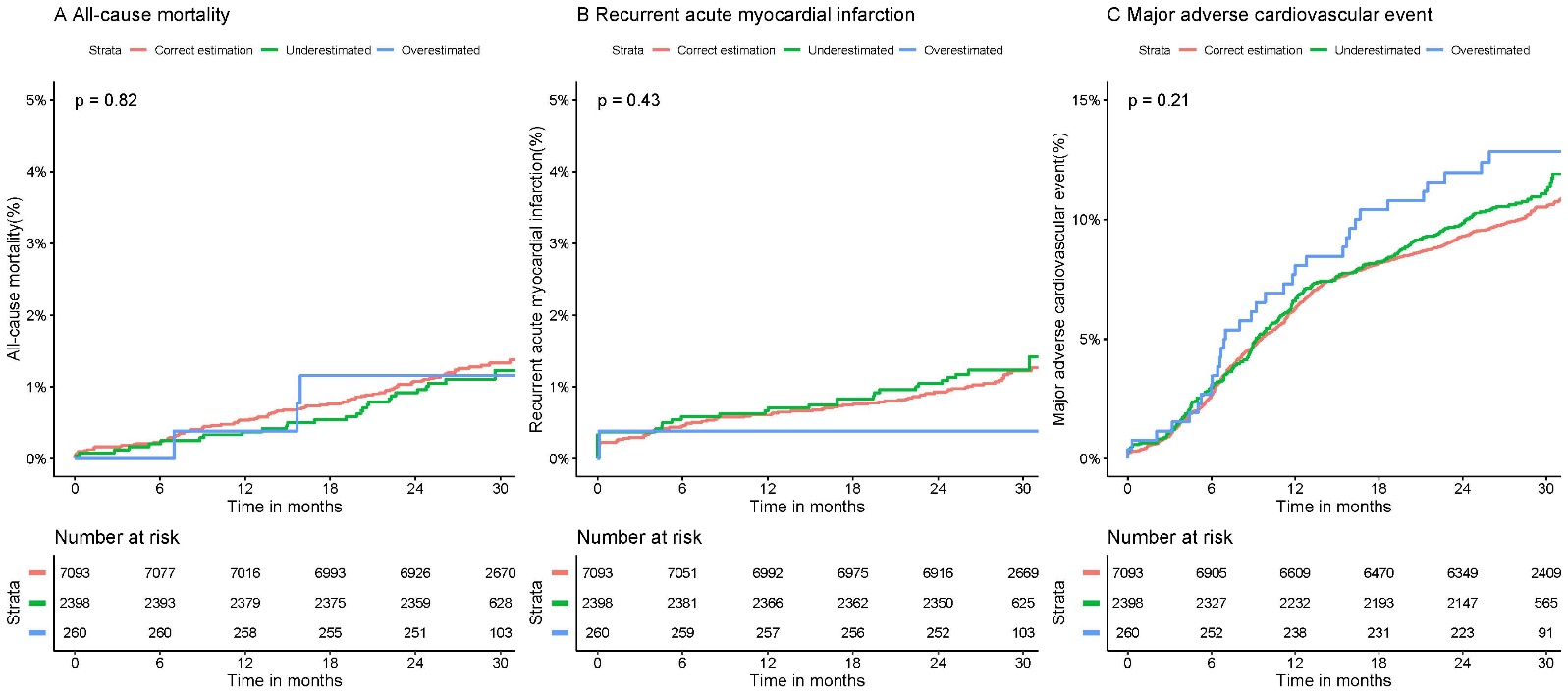
Supplemental Figure 6 Kaplan-Meier curves for 30-month adverse event rates in different classification of low-density lipoprotein cholesterol by the Friedewald equation. Overall rates of (A) all-cause mortality, (B) recurrent acute myocardial infarction, and (C) major adverse cardiovascular event.**

**
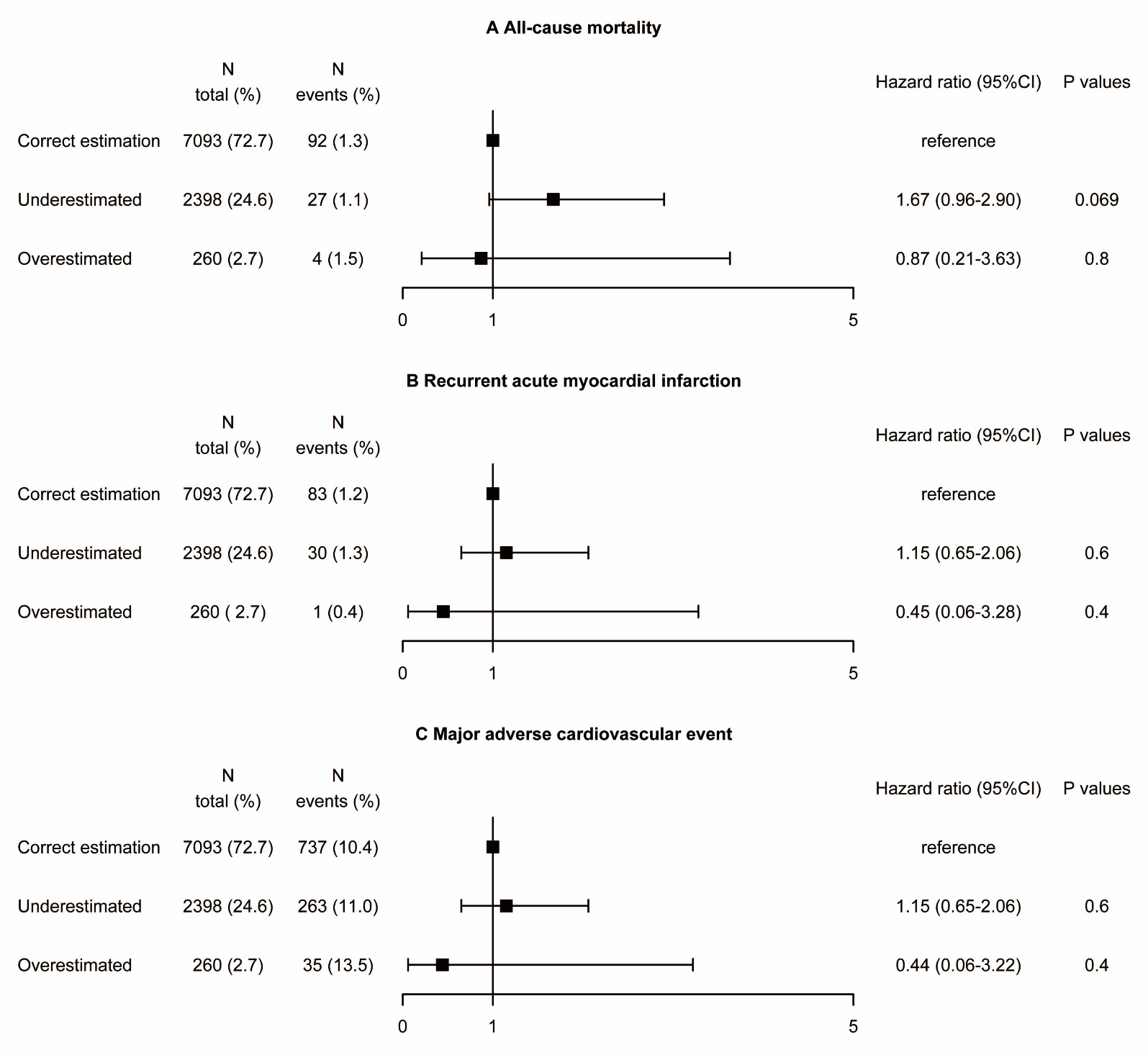
**

**Supplemental Figure 7 Association between misclassification by the Friedewald equation and the risk of (A) all-cause mortality, (B) recurrent acute myocardial infarction, and (C) major adverse cardiovascular event by multivariate analysis.**

Hazard ratios were from Cox proportional hazards regressions with multivariable adjustment for age, sex, current smoker, estimated glomerular filtration rate<60ml/min, complete revascularization, body mass index, ejection fraction, hypertension, and diabetes mellitus. CI = confidence interval.

**
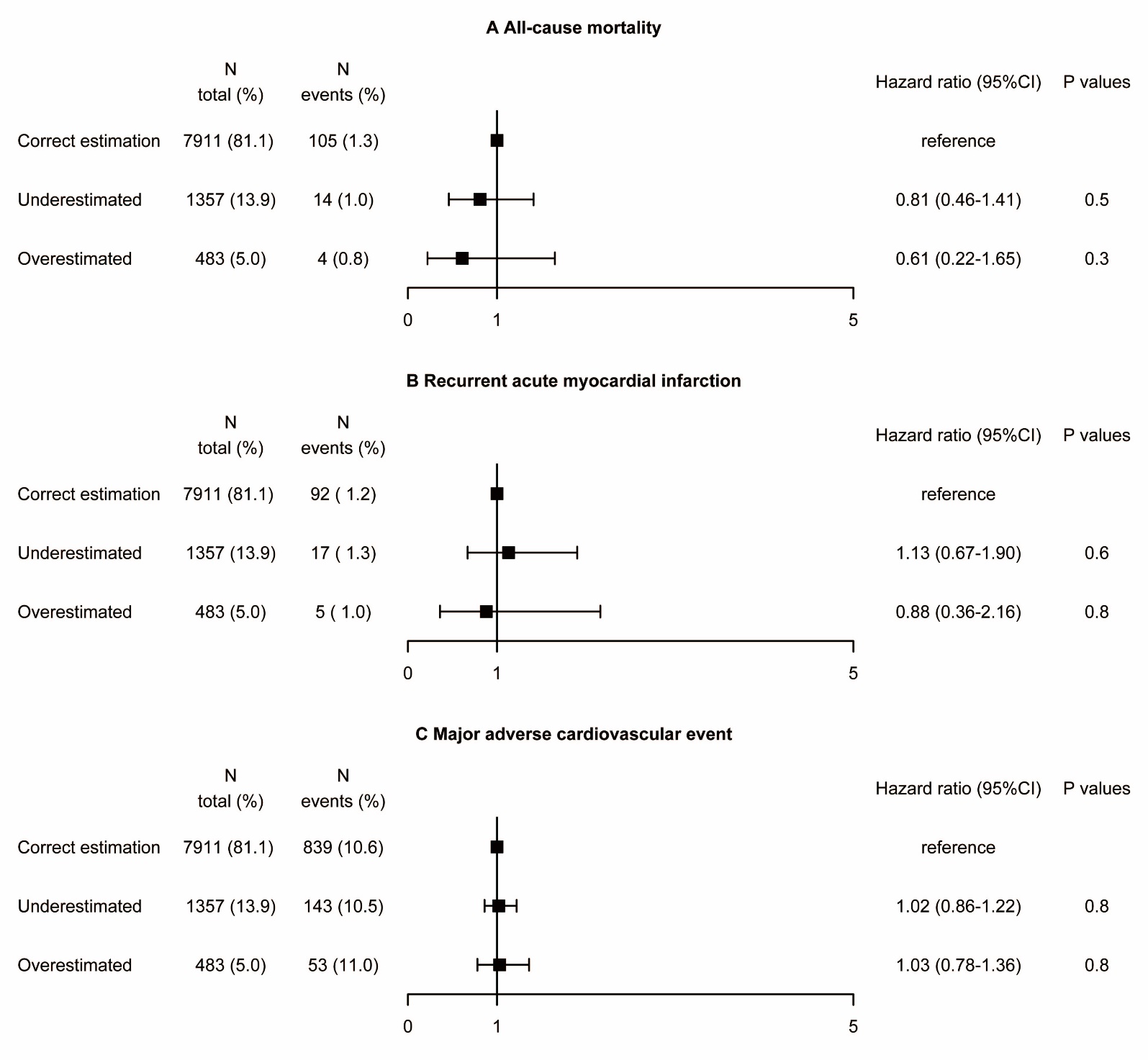
Supplemental Figure 8 Association between misclassification by the Sampson equation and the risk of (A) all-cause mortality, (B) recurrent acute myocardial infarction, and (C) major adverse cardiovascular event by univariate analysis.**

Hazard ratios were from Cox proportional hazards regressions with univariable analysis. CI = confidence interval.

**
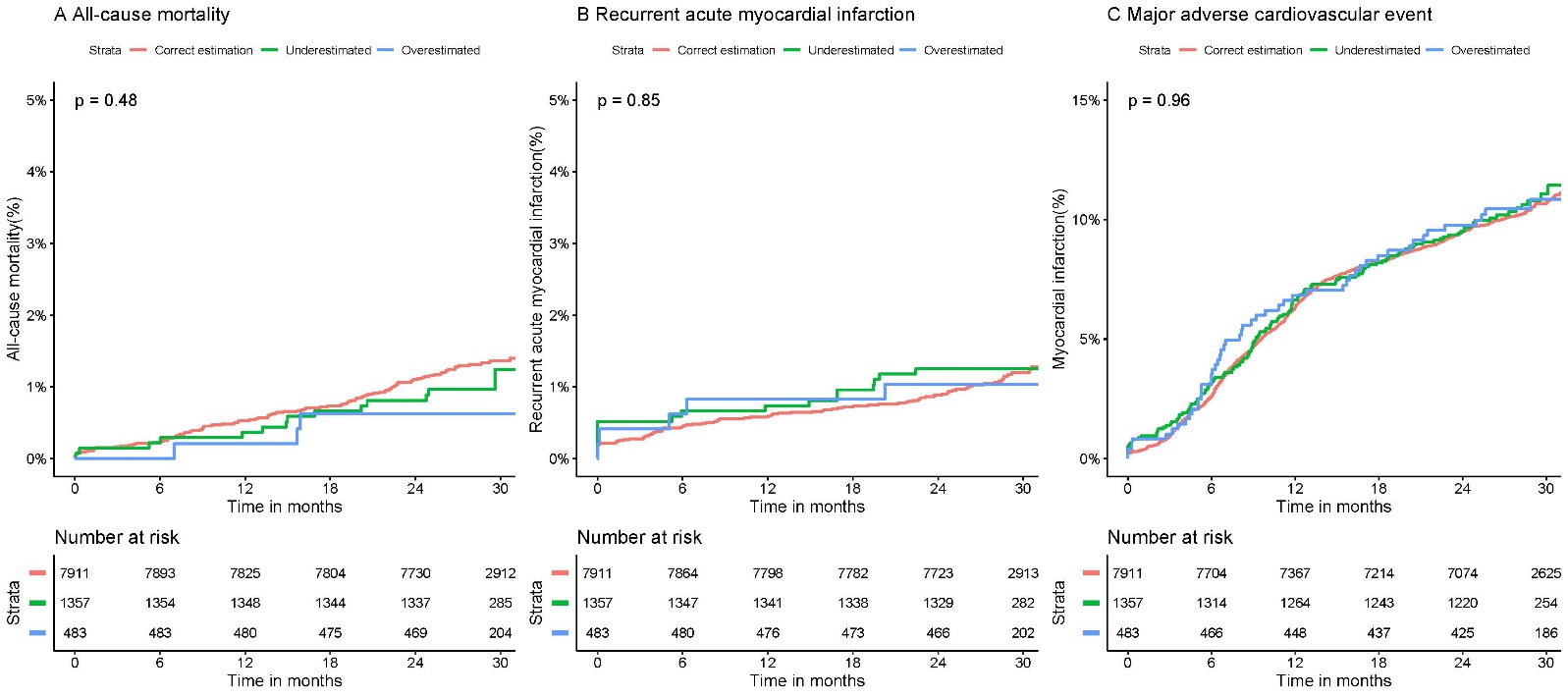
**

**Supplemental Figure 9 Kaplan-Meier curves for 30-month adverse event rates in different classification of low-density lipoprotein cholesterol by the Sampson equation. Overall rates of (A) all-cause mortality, (B) recurrent acute myocardial infarction, and (C) major adverse cardiovascular event.**

**
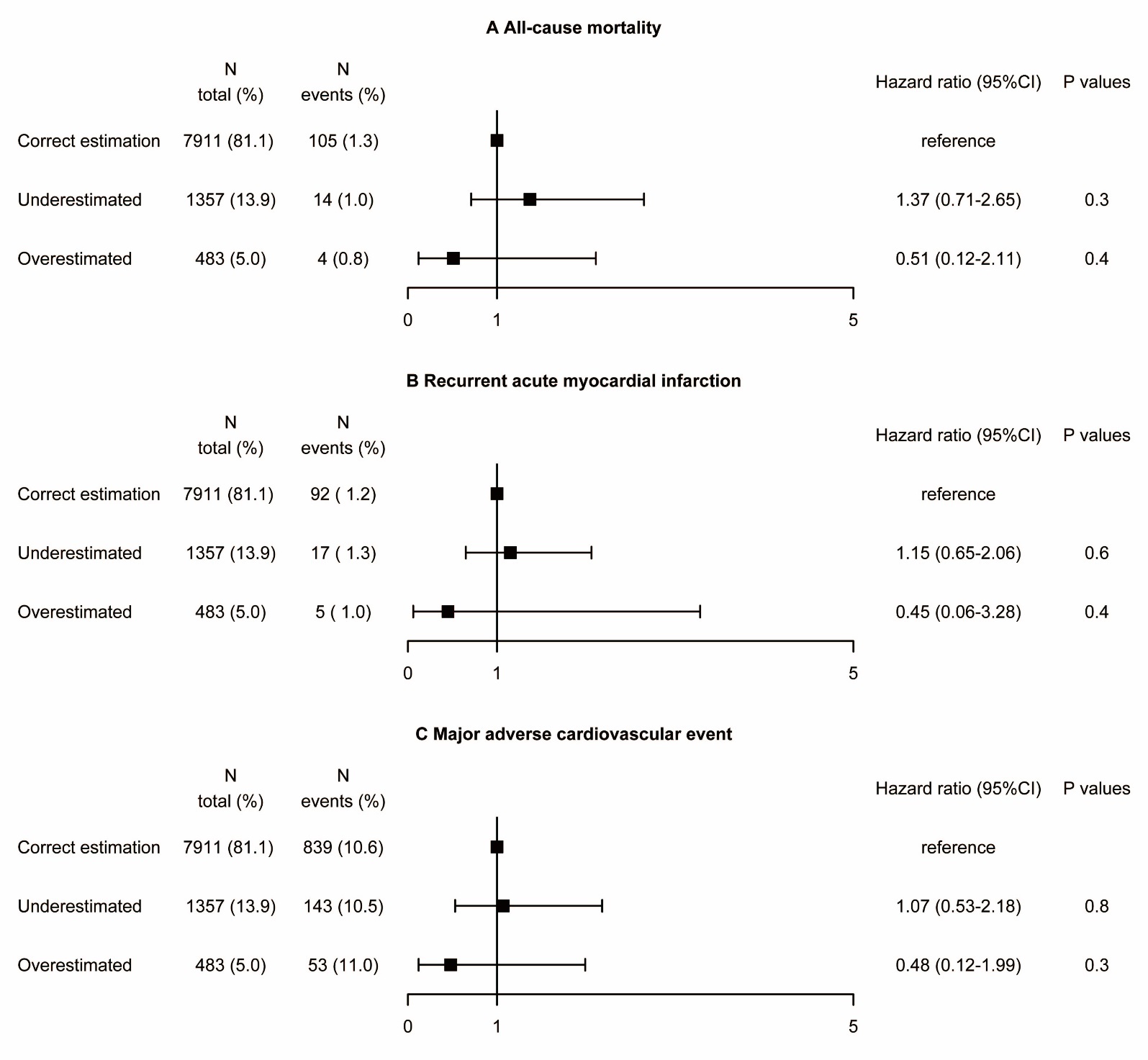
**

**Supplemental Figure 10 Association between misclassification by the Sampson equation and the risk of (A) all-cause mortality, (B) recurrent acute myocardial infarction, and (C) major adverse cardiovascular event by multivariate analysis.**

Hazard ratios were from Cox proportional hazards regressions with multivariable adjustment for age, sex, current smoker, estimated glomerular filtration rate<60ml/min, complete revascularization, body mass index, ejection fraction, hypertension, and diabetes mellitus. CI = confidence interval.


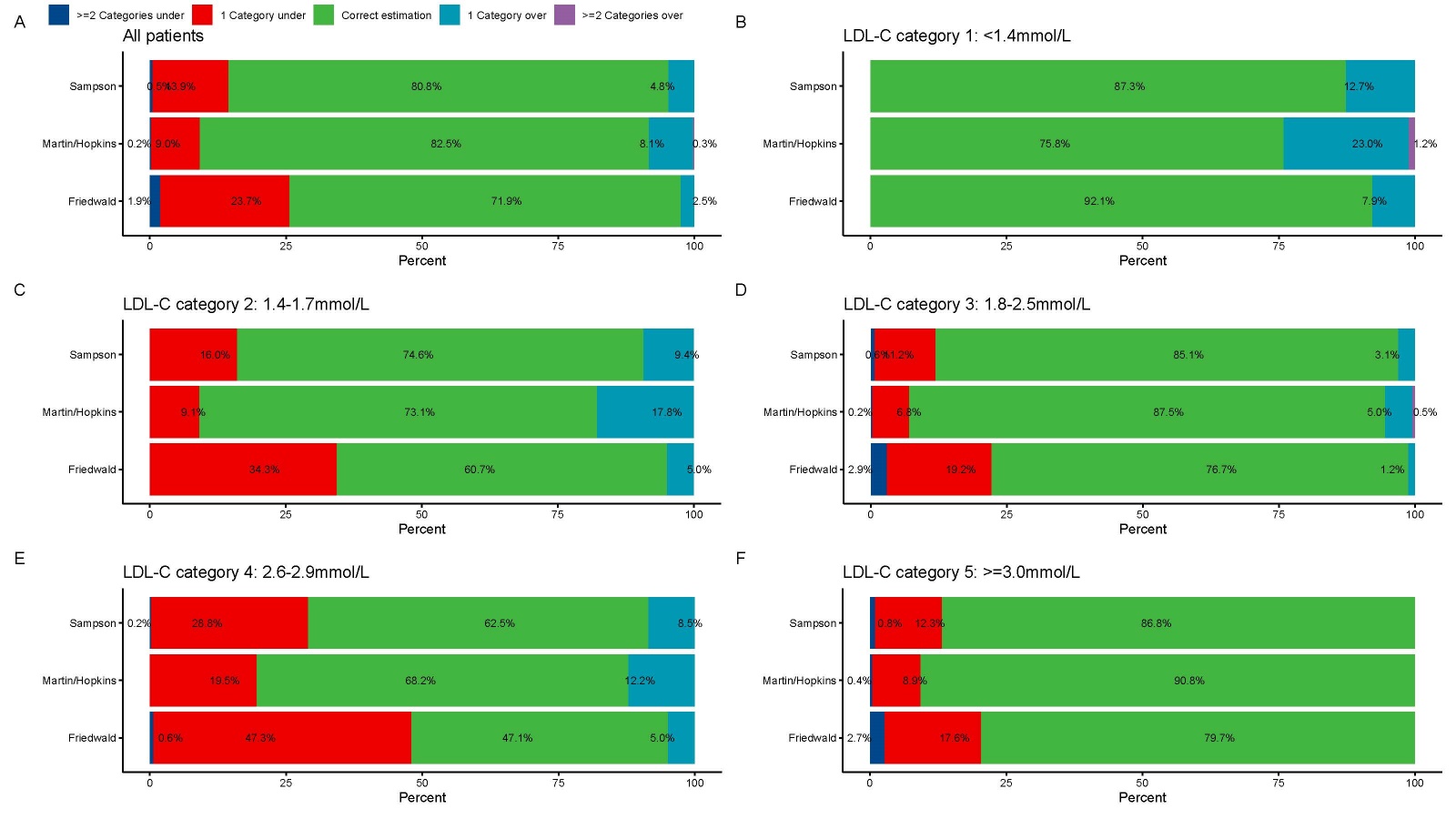


**Supplemental Figure 11 Proportion of misclassified patients per direction by estimated LDL-C category in patients with diabetes.**

Graphs represent the total percentage of under classified and overclassified patients within each LDL-C category. Values to the left and right of 0 on the x-axis indicate percentage under classified and percentage overclassified, respectively. Proportion of misclassification in (A)all patients, (B) LDL-C category 1: <1.4 mmol/L, (C) LDL-C category 2: 1.4-1.7 mmol/L, (D) LDL-C category 3: 1.8-2.5 mmol/L, (E) LDL-C category 4: 2.6-2.9 mmol/L, and (F) LDL-C category 5: >=3.0 mmol/L. LDL-C = low-density lipoprotein cholesterol.
